# Supplementary material for: Gaps in tropical science from unrepresentative distribution of sampling and citation across natural terrestrial environments
Source: Nat Commun. 2025 Dec 20;16:11378. doi: 10.1038/s41467-025-67617-4 (PMC12727867; doi:10.1038/s41467-025-67617-4)
Supplement: Supplementary file 1 — Supplementary Information [file 41467_2025_67617_MOESM1_ESM.docx]

**Supplementary information**

*
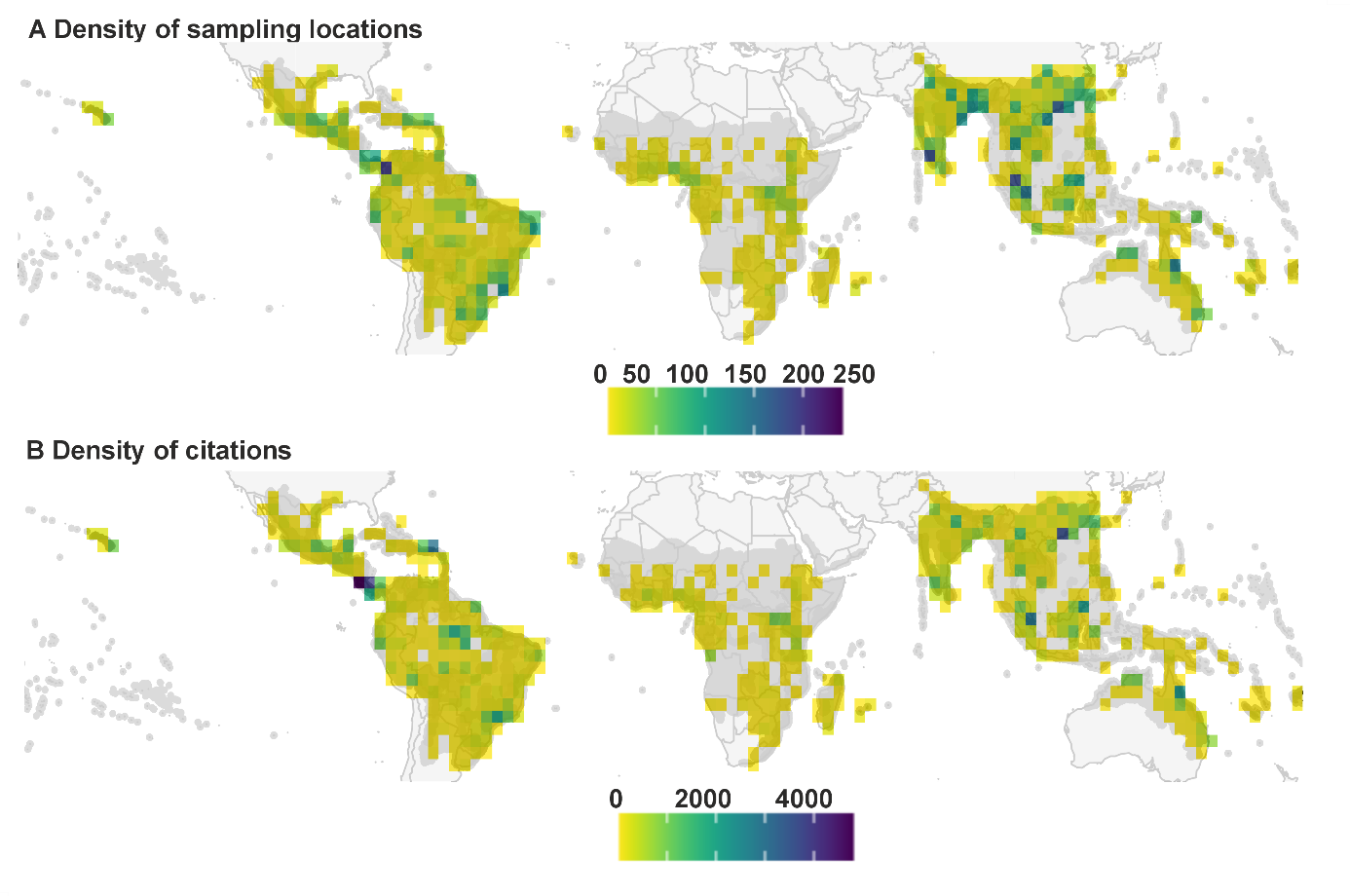
*

**Supplementary Fig. 1. *Density of sampling locations (A) and citations (B) per unit land area across all terrestrial habitats in the tropics.*** *Spatial resolution is 3° (~ 330 km). Maps were produced from a database of 4 260 articles, representing 9 987 sampling locations and 131 030 citations. The full extent of tropical biomes is highlighted in dark gray, using widely accepted boundaries****^23^****. To account for transition zones between the biomes, we added a buffer of 100 km around the formally defined tropical area. Overall, the study area consisted of 52.9 x 10^6^ km^-2^ of terrestrial land (ca. 36 % of the global land area). Base map from Natural Earth****^98^****.*


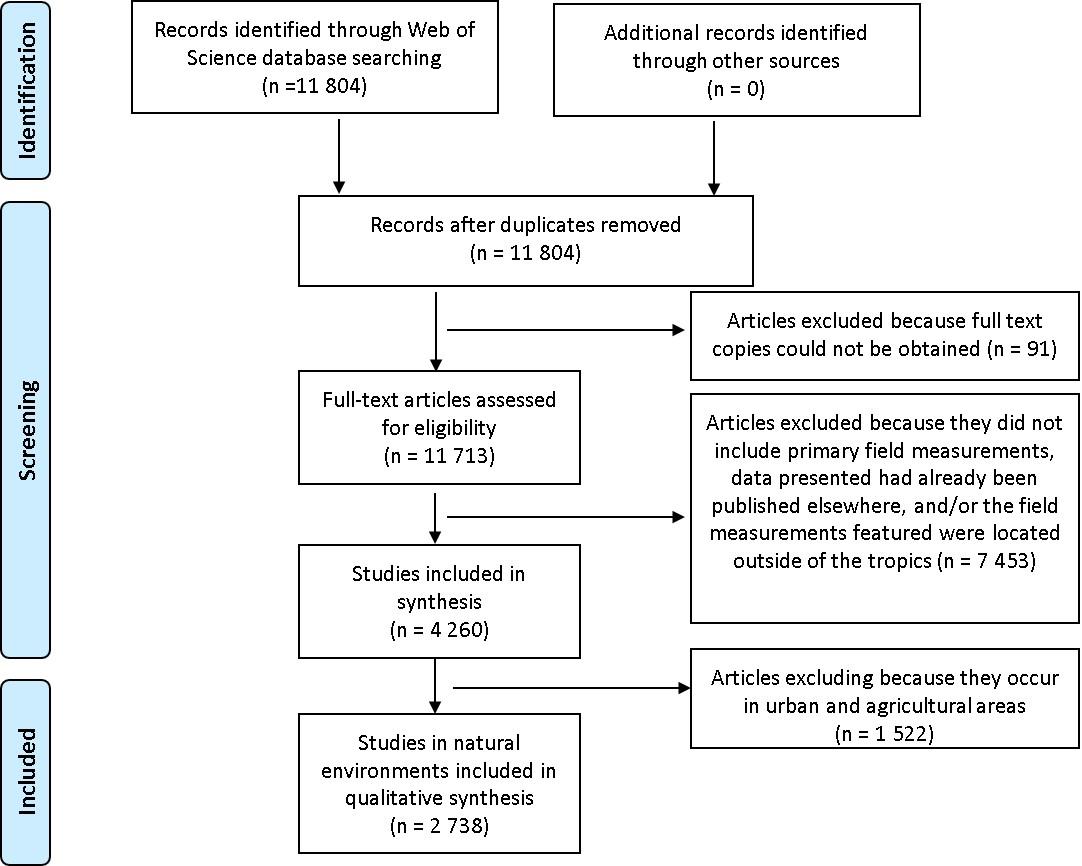


**Supplementary Fig. 2. *Flow diagram describing key steps in the literature review*.**


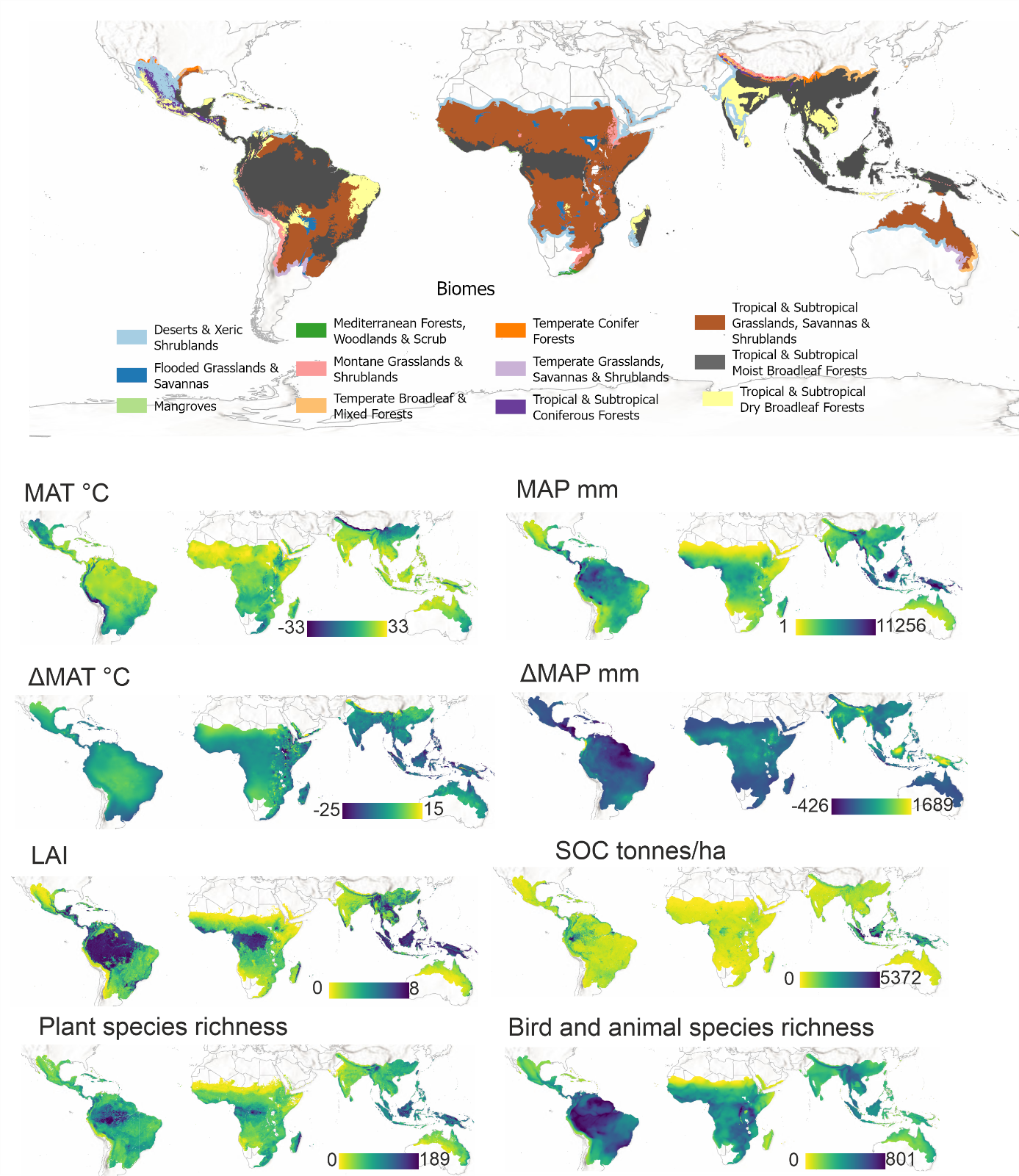


**Supplementary Fig. 3. *The tropical biomes^23^ and environmental conditions used in the study^27-31^.*** *Note that in most layers, the minimum and maximum values can be extremely low or high, but these extremes are not widespread across the area. Base map from Esri****^99^****.*

| **Ecoregion** | **Credit** | **License** | **Link** |
| --- | --- | --- | --- |
| Guinean mangroves | Julien Saison | Creative commons -share alike | https://commons.wikimedia.org/wiki/File:Cueilleuse_traditionnelle_d%27Hu%C3%AEtres_de_mangrove,_delta_du_Sine_Saloum,_femme_du_village_de_Soucouta,_S%C3%A9n%C3%A9gal.jpg |
| Ethiopian montane moorlands | Nina R | Creative commons -generic | https://commons.wikimedia.org/wiki/File:Bale_Mountains_National_Park_(49849653091).jpg |
| Southeast Tibet shrublands and meadows | NoGhost | Creative commons -share alike | https://commons.wikimedia.org/wiki/File:Southeast_Tibet_meadows.jpg |
| Marianas tropical dry forests | CT Snow | Creative commons -generic | CC-Generic  https://commons.wikimedia.org/wiki/File:Rota_Island_in_the_Commonwealth_of_Northern_Mariana_Islands.jpg |
| Purus-Madeira moist forests | Erick Caldas Xavier | Creative commons -share alike | CC-Share alike  https://commons.wikimedia.org/wiki/File:Floresta_Nacional_do_Iquiri_Erick_Caldas_Xavier_(6).jpg |
| Borneo lowland rain forests | Mike Prince | Creative commons -generic | CC-Generic  https://commons.wikimedia.org/wiki/File:Dipterocarp_Forest_at_Danum_Valley_(13997709808).jpg |
| Uatumã-Trombetas moist forests | Ministério da Ciência, Tecnologia e Inovação | Creative commons -generic | https://commons.wikimedia.org/wiki/File:A_(12)_(20932801935).jpg |
| Puerto Rican moist forests | Alessandro Cai | Unrestricted | https://commons.wikimedia.org/wiki/File:Rain_Forest_of_El_Yunque,_Puerto_Rico.jpg |
| Jian Nan subtropical evergreen forests | SuTa-mei | Creative commons -share alike | https://commons.wikimedia.org/wiki/File:A_forest_scene_of_Xibin_town,Youxi_county.JPG |
| Isthmian-Atlantic moist forests | MongeNajera | Creative commons -share alike | https://commons.wikimedia.org/wiki/File:Braulio_Carrillo_National_Park_2.jpg |

**Supplementary Table 1. *Details of photographs used in Figure 2 in main manuscript.*** *The photographs were modified from the originals. Use of these photographs does not constitute endorsement by the licensor.*
