# Supplementary material for: Gaps in tropical science from unrepresentative distribution of sampling and citation across natural terrestrial environments
Source: Nat Commun. 2025 Dec 20;16:11378. doi: 10.1038/s41467-025-67617-4 (PMC12727867; doi:10.1038/s41467-025-67617-4)
Supplement: Supplementary file 2 — Description of Additional Supplementary Information [file 41467_2025_67617_MOESM2_ESM.pdf]

Description of Additional Supplementary Information

Supplementary Data 1

Sampling and citation data for the full list of ecoregions included within the study area. The study area includes both accepted boundaries for tropical biomes[23], and a buffer of 100 km around this formally defined tropical area.
